# Mobile Intervention for Individuals With Psychosis, Dual Disorders, and Their Common Comorbidities: A Literature Review

**DOI:** 10.3389/fpsyt.2019.00302

**Published:** 2019-05-03

**Authors:** Antoine Pennou, Tania Lecomte, Stéphane Potvin, Yasser Khazaal

**Affiliations:** ^1^Schizophrenia and Psychoses Study Laboratory for Intervention and Recovery, Department of Psychology, University of Montreal, Montreal, QC, Canada; ^2^Research Center of the Montreal Mental Health University Institute, Intervention Axis and Services & Neurobiology and Cognition, Mental Health University Institute of Montreal, Montreal, QC, Canada; ^3^Addiction Medicine, Department of Psychiatry, Lausanne University Hospital, Lausanne, Switzerland

**Keywords:** severe mental illness, dual disorders, schizophrenia, psychosis, substance use disorder, mental health apps, emotion regulation

## Abstract

Over 50% of people diagnosed with a severe mental illness, such as schizophrenia or bipolar disorder, will meet criteria for a substance use disorder in their lifetime. This dual disorder often starts during youth and leads to significant societal costs, including lower employability rates, more hospitalizations, and higher risk of homelessness and of suicide attempts when compared to those with a serious mental illness without substance misuse. Moreover, many individuals presenting with comorbid disorders also present with other psychological difficulties as well, such as personality disorders or anxiety and depression, also known as complex comorbid disorders. Transdiagnostic treatments that focus on core difficulties found in people with complex dual disorders, such as emotional regulation, are direly needed. Emotional regulation skills can help reduce distress related to psychotic symptoms and maintain abstinence in substance use disorders. New technologies in the field of communications have developed considerably over the past decade and have the potential to improve access to such treatments, a major problem in many health care settings. As such, this paper aims at: presenting core difficulties present in many individuals with dual disorders, reviewing the scientific literature pertaining to the use of mobile applications in mental health and addictions, and presenting the development and potential of a new application for emotional regulation for people with dual disorders.

## Introduction

Severe mental illness (SMI) typically includes psychotic episodes and can be characterized by the presence of positive (e.g., delusions, hallucinations) symptoms, but also negative or depressive symptoms (e.g., flat affects, avolition) or symptoms of disorganized thought and behavior (1). It is also in this category that bipolar disorders are grouped, which are characterized *inter alia* by the alternation between depressive and manic or hypomanic episodes (i.e., irritable or abnormally high mood). It is estimated that about 3.5% of the population will be diagnosed with an SMI at some point in their lives—the highest incidence reported or recorded being between 15 and 30 years (2). SMI is also characterized by mild to moderate cognitive deficits in the majority of individuals (25% of them will maintain a cognitive functioning within the norm) (2). Although they have traditionally received less attention than the symptoms associated with psychosis (e.g., positive symptoms), these features are among the most important determinants when it comes to estimating the patient’s capacity to function autonomously in his/her environment (2, 3).

SMIs are marked by social functioning difficulties (more specifically difficulties starting or maintaining a conversation, attaining a goal, or meeting basic needs requiring a simple conversation/interaction with others). Furthermore, these difficulties will lead to social integration problems that can occur throughout different stages of the disorder (2). Studies report that the fear of being stigmatized and the fear of being judged (the latter often linked to social phobia) are two of the most frequently reported obstacles to integration by participants (4). Because of its significant influence on an individual’s social integration capacity, an increasing number of interventions focus first and foremost on the individual’s functional capacities rather than solely on the reduction of symptoms (5).

In terms of clinical presentation and important difficulties people with SMI face, substance misuse needs to be addressed. Indeed, over half of people with an SMI will also have a lifetime substance use disorder (2, 6–8). A hypothesis that might explain this phenomenon is that individuals with SMI have a deficit in the reward circuit (i.e., neural network involving the ventral tegmental area and the nucleus accumbens) (9). This would make individuals with SMI sensitive to the gratifying effects of drugs, and so, it would be more difficult for them to resist the urge to consume (10, 11). However, it has been documented that people with SMI will use substances for similar reasons as those of people without SMI use, such as to feel more socially comfortable, to relax, to fight boredom, to feel euphoric, etc. Among the consequences related to dual disorders (i.e., SMI + substance use disorder), we find a higher rate of violent crimes (12, 13), more hospitalization, poorer compliance to treatment (13, 14), more relapses (14), a higher rate of sexually transmitted and blood-borne infections (STBBIs, including HIV) (15), a higher rate of homelessness (16, 17), more psychiatric symptoms (14, 18), poorer treatment outcomes (19), and a greater suicide rate (20) than people with a noncomorbid SMI.

## Dual Disorders and Common Comorbidities

Concurrent disorders are often accompanied by other issues that complicate the clinical presentation. SMI is unfortunately recognized to be associated with a greater prevalence of traumatic experiences. One in three patients with SMI currently undergoing treatment has a post-traumatic stress disorder (PTSD) (2). In dual disorders, the rate of comorbid PTSD and traumatic experiences is 49% and 73%, respectively. Traumatic experiences in individuals with psychotic disorders are related to more pronounced symptoms, increased use of services, more substance use, increased incidence of physical illness, and increased high-risk behaviors (2). Group B personality disorders (i.e., histrionic, borderline, narcissistic, and antisocial) are known for emotional instability, the tendency to dramatize certain situations, and unpredictable behaviors (1). A significant portion of these individuals struggle with substance use issues. An epidemiological study reports that 78% of individuals with borderline personality disorder met the criteria for substance use disorder at least once in their lifetime (21). Individuals with group B personalities are known to show more impulsive behavior, greater mood swings, and more depressive symptoms (22). In a study on psychotic disorders, a significant proportion of patients (close to 30%) diagnosed with SMI displayed problematic personality traits associated with group B without necessarily being diagnosed with a personality disorder. Wickett et al. (23) reported the presence of these traits in 27 (59%) of the 46 individuals enrolled in their studies [the presence of traits associated in this study was determined by the MCMI-III score (i.e., score between 75 and 84)].

Fifty percent of people recently diagnosed with SMI report experiencing depressive symptoms (24). In dual disorders, a study of the different clinical correlates in SMI with data from the *National Institute of Mental Health* (NIMH) in the United States (n=1,460) mentioned that 32.2% of individuals with concurrent disorders reported having a major depressive episode in the 5 years preceding the study (18). Substance use in concomitant disorders is associated with more severe symptomatology than noncomorbid SMI (25). There is a much higher rate of completed suicides in SMI compared to the general population (147 to 750 deaths per 100,000 persons compared to 11 per 100,000 per year) (2).

## Emotion Regulation and Psychosis

The study of emotional regulation is mainly based on two predominant conceptual frameworks (26). The first, Gross’s (27, 28) model, defines emotion regulation as the use of different strategies to increase, decrease, or maintain an affective experience and its expression. This model is mainly used when trying to establish the usefulness and/or effectiveness of specific emotion regulation techniques in psychiatric symptoms (26). The second model, presented by Gratz and Roemer (29), is more integrative. It addresses emotion regulation in the context of the general affective functioning of the individual, subdivided into four dimensions: 1) the awareness, understanding, and acceptance of affective experience; 2) the ability to engage in a goal-oriented activity and restrain from acting impulsively when experiencing negative emotions; 3) the ability to use techniques that are appropriate to the context in which one finds himself/herself to modulate the emotional response; and 4) the openness to feeling negative emotions when pursuing a meaningful activity. According to this model, an individual having difficulty regulating his/her emotions will thus present difficulties in one or more of these dimensions (26). Studies using this model report that difficulties in all four dimensions are frequently observed in depressive, anxiety, eating, substance use, and borderline personality disorders (26), some of which are frequently associated with SMI (see the section Dual Disorders and Common Comorbidities).

Emotion regulation difficulties have also been identified as one of the explanatory factors for substance use in schizophrenia. In their model, Blanchard et al. (30) suggest that individuals with certain personality traits (i.e., negative affectivity/neuroticism and disinhibition/impulsivity) who also show difficulties in applying effective coping methods (e.g., interpersonal problem-solving) are more at risk of using substances to cope with negative emotion when encountering important stresses. This model has been partly supported by the literature (31).

Studies have shown that the use of good emotion regulation strategies leads to a reduction in the intensity and distress associated with auditory hallucinations (32, 33). It would also lead to a decrease in the likelihood of developing psychotic symptoms and prescribing medication in “high-risk” individuals (34) and a reduction in the relapse rate (35). Emotion regulation difficulties are central in comorbidities associated with SMI. Emotional regulation difficulties in individuals diagnosed with PTSD would be associated with, among other things, difficulties in functioning and more pronounced symptomatology (36). A study conducted by Ehring and Quack (37) on individuals with PTSD (n = 616) demonstrated that the severity of trauma-related symptoms is negatively associated with participants’ emotional regulation abilities (i.e., greater difficulties = more severe symptomatology). The authors of the study also point out that there are no differences between individuals with complex PTSD (i.e., repeated abuse, often starting in childhood) and those experiencing “one” trauma, with regard to their abilities to regulate their emotions. These difficulties are also a central issue in group B personality disorders (38) and substance use disorders (39). Indeed, many individuals will use substances in an attempt to cope with difficult emotions, as an avoidance strategy.

Today, more and more authors place a strong emphasis on emotional processes in the treatment of SMIs such as schizophrenia (40). This phenomenon may be explained in part by the fact that the level of distress experienced is proportional to the intensity of psychotic symptoms reported (41). Studies on emotion regulation within SMI have focused primarily on the use of two cognitive strategies—suppression and re-evaluation. Suppression is a strategy where the individual tries to voluntarily limit the expression of an emotion when it occurs; it is categorized as a regulation technique centered on the emotional response. Re-evaluation is a technique in which the individual tries to modify the meaning of an event in order to change the emotional response; it is a strategy centered on the antecedents of the emotional response (i.e., before feeling the emotion) (42). As for the suppression strategy, two diverging trends seem to emerge from the studies. The explanatory model of blunted affects by Kring and Werner (43) presents this manifestation as resulting from difficulties in increasing the affective experience and a less adapted use of regulation strategies. Following this idea, researchers have identified the use of suppression strategies as one of the potential explanations of blunted affect (42, 44, 45). Other studies report that participants with SMI use suppression as much as control participants (42, 46). Studies seem more consistent with the fact that people with SMI use fewer reassessment strategies than the average person (45, 46). This strategy is known to be associated with a reduction in depressed mood and better social functioning (42). The emotional processes observed in substance use disorders suggest that the search for stimulation through the consumption of substances would aim to lessen the experience of anhedonia, an often-reported state in this population (47, 48). We can also find more recent results going in the same direction where a study reported positive correlations between novelty research, craving, and anhedonia in patients previously dependent on opioids or alcohol (49). Those correlations have been reproduced through several studies; research on the subject identifies, in a somewhat paradoxical way, that the experience of anhedonia may be a consequence of substance use (48).

Emotion regulation is central to third-wave cognitive behavioral therapy approaches (50). These therapies often encourage the use of mindfulness and appear to have encouraging results in terms of improving emotion regulation when employed with individuals with psychotic disorders (51) as well as those with substance use disorders (52). Among these approaches, some of the better known are dialectical behavior therapy, mindfulness therapy, and acceptance and commitment therapy (ACT). Dialectical behavioral therapy is an intervention initially intended for individuals with borderline personality disorder with a suicidal tendency; it was subsequently adapted to all issues involving emotional regulation difficulties (28). This therapy proposes strategies for emotion regulation according to six dimensions: 1) emotional vulnerability factors; 2) events (internal/external) leading to an emotional response; 3) interpretation of events; 4) the emotional response (physiological, cognitive, experiential aspects)…; 5) the verbal/nonverbal response and the actions taken; and 6) the impacts of the emotion felt (e.g., secondary emotion) (53). Mindfulness-based therapy is an intervention aimed at developing awareness of the present moment by taking an attitude of nonjudgment (28). Finally, ACT promotes the acceptance emotional states in order to help individuals commit to goals in accordance with their values (54). Studies support that this intervention is associated with an improvement in emotion regulation and a decrease in anxiety and depression symptoms for those with early psychosis (51) as well as those with SMI with comorbid traumatic childhood experiences (55). Third-wave treatment among users of different substance categories also produced encouraging results in which there was a significant reduction in drug use but also a better retention than in treatment as usual (56). Interventions, such as third-wave treatments, that offer better emotion regulation strategies to people with comorbid disorders, namely SMI with substance misuse, are needed and could likely improve the individuals’ lives not only by diminishing the need to use drugs to cope but also by helping decrease distress, associated with various clinical presentations.

## Access to Treatment

Difficulties in accessing mental health services are a major global concern, particularly in relation to drug and alcohol addiction issues (57, 58). This situation is even more concerning given the link between difficulties in receiving services and the onset use of injectable drugs among individuals already experiencing substance use disorders (59). In order to better target accessibility needs, Lesvesque and colleagues (60) suggest conceptualizing access to services in five dimensions, derived from a synthesis of the literature on the subject: accessibility, acceptability, availability, costs, and relevance of services. Accessibility refers to the importance of services being known to the population. This is particularly important considering findings that suggest that individuals with substance abuse disorders are more likely to favor familiar treatments (61). Acceptability refers to the cultural and social dimensions that must be compatible with the type of service offered (e.g., belief in the cause and treatment of diseases). Availability refers to the resources required for a facility to provide services to the entire population and to be “physically” accessible (e.g., within-territory distribution based on the density of population, flexibility of work schedules, and adaptation of the environment for people with reduced mobility). Service costs refer to the capacity of individuals to spend money in order to receive a service. Finally, the dimension of relevance of services highlights the importance of matching services to the needs of clients.

Issues regarding the availability of services seem to be one of the main barriers to access in addiction issues, particularly in relation to the problem of waiting time (59, 61–65). Studies on this matter conducted among individuals with dual disorders also report difficulties in the relevance of care provided in this population, who, when compared to individuals with a unique diagnosis of substance use disorder, appear as those being the least satisfied with services rendered due to a lack of compatibility with their needs (e.g., treatment setting for a specific disorder that doesn’t accept people with substance use disorder) (66). Acceptability of care has also emerged as an important issue in this population—with the majority of individuals indicating that they prefer to manage their symptoms independently (66). In addition, individuals with dual disorders often report feeling excluded from health care services because of the dual diagnosis (mental health services do not accept them because of substance use disorder and vice versa). A preference for more personalized services, such as integrated treatments, was mentioned along with detailed reports regarding the lack of perceived usefulness of a parallel treatment focusing on a single diagnosis at a time (67).

Canadian studies on access to services seem to raise the same concerns as elsewhere in the world with a few exceptions. As the public health system bears the costs of certain interventions such as the use of opioid antagonists, it is possible that issues related to the cost of certain services may be less significant than in other countries lacking a similar structure (64). In Ontario, a report on the quality of mental health and addiction services states that individuals unable to access services reported problems of availability (i.e., difficulties with language, service hours in conflict with working hours), accessibility (i.e., not knowing where/how to get help), costs related to services (e.g., insurance does not cover certain services), and acceptability (e.g., fear of stigma related to the request for help) (62). In Quebec, a study by Champagne and Contandriopoulos (68) on the impact of the recent reorganization of health care services between 2015 and 2017 reveals a number of disturbing findings regarding the accessibility of care in mental health and addiction treatment (68). Based on the information collected in the integrated health and social service centers [Centres Intégrés de Santé et de Services Sociaux (CISS)], the organizations in charge of providing services in the region, researchers found that access to mental health services remains problematic and inadequate for the whole region (i.e., less privileged centers offer fewer services than more privileged centers). With regard to access to addiction services, researchers point out that although several centers have recently integrated detection and early intervention services, only one CISSS (center) had a satisfactory accessibility score (i.e., 79% of patients had been consulted within 15 days).

## The Smartphone, an Increasingly Popular tool

### Assessment

We have witnessed a significant increase of people with cell phones from 2010 to 2015 (69). This phenomenon is observed in several age groups, different socioeconomic levels, and several different cultures from around the world (70). A study of patients attending hospital emergency departments reported that 70% (176/249) of individuals struggling with homelessness in the past year had a cell phone (or a smartphone—76/176, 43.2%) in their possession (71). In its annual report on the issue, the United Nations–sponsored international telecommunications union estimates that 90.3% of people in developed countries have a subscription to a mobile phone service (72). With regard to people suffering from a psychotic disorder, a survey assessing 1,592 individuals with a serious mental illness (i.e., schizophrenia, schizoaffective disorder, and bipolar disorder) reported that 72% of participants had a smartphone (73). The smartphone adaptation of mental health interventions offers several advantages that can potentially mitigate certain problems encountered by the community. It could allow the assessment of individuals in their natural environment, where some mental health issue originally emerged, instead of studying them in more artificial contexts such as a clinic or a hospital (5). Furthermore, by allowing someone to assess his/her current state (see section below) and to access support at any time (and any place) needed, this new medium could be part of the answer to treatment accessibility issues (74).

Data collection using applications can be organized in different ways. Among these approaches, the ecological momentary assessment (EMA), defined as “methods using repeated collection of real-time data on subjects’ behavior and experience in their natural environments” (75, p. 3), appears as a promising method in the use of new technologies in mental health and addiction (70). Shiffman et al. have published a comprehensive record on the issue in the 2008 *Annual Review of Clinical Psychology* (75). The authors report that this method makes it possible to overcome the important stakes of memory bias (e.g., memory salience depending on emotional valence), which researchers are necessarily confronted with when relying on retrospective evaluation of a phenomenon occurring in the natural environment of the subject. They also mention that this method makes it possible to capture more precisely the variations of a phenomenon over time (for example, mood levels during the week evaluated twice a day) because it makes repeated measurements of the phenomenon in a relatively small time gap. These two factors contribute to increasing the ecological validity of the EMA (70, 76, 77). This greater validity could potentially help better understand the “risk situation–feelings of craving–consumption of substances” sequence in substance use disorders (78). Shiffman et al. (75) proposed four potential uses of EMA in research: identifying individual differences by comparing multiple groups, tracking the “natural history” of one or more topics to capture variations in phenomena across time, studying the interaction of two or more phenomena in an individual’s environment (e.g., mood levels following a stressful event), and studying the temporal sequence of certain phenomena (e.g., contextual or internal factors leading to substance use). By offering the possibility to perform assessments on a regular basis or according to the events under study, the EMA is flexible enough to adapt to the study of several psychological phenomena such as episodes of craving in substance use—an event occurring in a small time frame—or general mood that often requires multiple assessments (75). Much research in the field of mental health applications has incorporated this approach. Researchers working among adolescents have reported that using this method would produce valid results in the assessment of symptoms, environmental contexts, and coping strategies used in real time by users (79). A study by Comulada et al. (80) including adolescents with substance use disorders (13 to 18 years old) was able to clarify the external and internal cues related to drugs and alcohol consumption. With the help of an application-based EMA, these researchers were able to establish that, compared to other drugs, alcohol consumption occurs mainly on evenings and weekends. In the field of SMI, research using this method has suggested a link between the environmental context and the intensity of hallucinations (81) and a greater reactivity to daily stresses between individuals at risk of developing psychosis (82).

### Intervention

Following the same principles as EMA, interventions using smartphone applications or ecological momentary intervention (EMI) are considered interventions in the individual’s everyday environment (83). Although the study of these interventions is still in its infancy, researchers have reported encouraging results for the use of EMI for several health issues, such as weight loss (84), reduction of anxiety levels (85), reduction in the number of cigarettes smoked (86), and increase in physical activity (87).

The National Institute of Mental Health (NIMH) in the United States identifies six categories of mental health apps (74):

- Applications designed to promote autonomous disease management that provide feedback to the user based on information previously entered.
- Applications to correct thinking biases (i.e., cognitive restructuring), mainly for people with psychotic disorders.
- Applications for skills training (e.g., stress management).
- Applications to get in touch with a mental health professional.
- Applications that automatically record indirect indicators believed to be related to a disease (e.g., number of text messages sent for a socialization index, duration of calls,…).
- Applications that collect multiple categories of data automatically.

Growing evidence tends to support these different applications as effective methods of prevention and intervention (70). Among other things, these apps have already been associated with a reduction in depressive symptomatology and anxiety and an improvement in substance use problems in several studies (88). However, it is important to note that this field of research is still young; the majority of research published in this field are pilot studies with no comparison group or randomized studies [Randomized controled trials (RCTs)] that have not yet been replicated (89). Recent research has also reported that this intervention format is well received by adolescents dealing with mental health issues (90, 91) or physical health issues (92).

In the field of SMI, a literature review by Firth and Torous (93) identified five applications, evaluated in seven separate articles—those apps were primarily designed to promote self-management of the disease. The authors report that the majority of applications were very well received by participants, with an estimated average use of 85% of the days in which the studies were conducted, 3.95 times per day. It also states that people at risk for a psychotic episode and those who just experienced their first psychotic episode were most likely to use the app. Another study involving 33 individuals with SMI or schizoaffective disorder assessed the feasibility and acceptability of the FOCUS app, an application offering the possibility of obtaining clinician assistance, and reported positive results going in the same direction (use: 86.5% of days, 5.2 times per day) (94). An original aspect of this study was that the design of the application took into consideration the cognitive impairments of the targeted population (95). A recent study by Schlosser et al. (96) has also reported encouraging results on the use of the PRIME application (i.e., personalized real-time intervention for motivational enhancement) among individuals who have recently experienced a first psychotic episode. Aimed at fostering goals for better health, quality of life, interpersonal relationships, and greater productivity, PRIME invited participants to select one of 36 life goals (e.g., improve my relationship with my family) that they wish to pursue. Short-term goals derived from life goals (e.g., doing a fun activity with a family member) were then suggested to the participant. The application also allowed users to send messages to experienced clinicians and other users of the application on an online platform for advice or support to achieve their goals. Researchers report that individuals who used the app over a 12-week period significantly improved their sense of self-efficacy and motivation and had a significant reduction in the severity of their depressive symptoms and defeatist thoughts (96).

Encouraging results have also been seen for applications targeting disorders that are often comorbid in SMI. In September 2018, the Food and Drug Administration (FDA) authorized the marketing of the first application designed to assist in the treatment of substance use disorders, the application reSET. It is the first application of its kind available only under medical prescription (97). The application consists of a contingency management module of 62 exercises aiming at enhancing personal well-being without resorting to substance use (i.e., how to improve communication, mood management, prevention of transmission of sexually transmitted infections, …). In order to facilitate monitoring, the user’s activity on the application is listed on a web page accessible to the physician (97, 98). The validation study of the application, performed with 507 people, demonstrated that the use of the application was associated with more days of abstinence and a lower rate of attrition than in the treatment-as-usual condition (98). It is important to mention that this study, which served as justification for the approval of the application by the FDA, was performed on a desktop-based intervention, which was subsequently adapted to the format of a mobile application (97, 98). In the adult cannabis-using population, a mobile app called Stop-cannabis was developed in Switzerland by one of the authors (YK) and colleagues and reported more than 73,000 users in 4 years (currently about 1,300 active users and 13,000 sessions per month). Based on the self-determination theory, this application aims to promote autonomy, competence, and relatedness as part of a process to reduce cannabis use. Following these principles, the various interventions offered by the application are based on brief intervention in substance use disorder, motivational interviewing, and the principles of relapse prevention for substance use disorder (99). More specifically, the application first gives access to a series of psychoeducation modules on the effects of cannabis withdrawal and suggests alternatives to consumption for a craving episode (e.g., call a loved one, entertain yourself on social networks). Following the principles of motivational interviewing, the application allows the user to identify the advantages and disadvantages of stopping cannabis use. The home page also displays several cues to facilitate cannabis cessation (i.e., the number of days without consumption, the amount of money saved due to the cessation of consumption, and the possibility of purchasing rewards after a certain number of days without taking cannabis). In 2016, an analysis of data related to 22,000 users showed that they compiled 722,000 app sessions, with more than 30% of people having more than 6 sessions. Furthermore, a subgroup of 10% of users had performed 20 or more sessions. In terms of satisfaction, around 70% of them reported daily use of the app, and 80% reported that the app helped them to stop or reduce cannabis consumption “a little” or “a lot” (99). The Stop-cannabis app is currently under validation, and preliminary results demonstrate its feasibility and potential for cannabis adult users wishing to stop using. This application combined with an EMA program is now under study with adolescents with dual disorders (100). Young cannabis users (mean age: 20 years) who followed two sessions of motivational enhancement therapy combined with a mobile intervention designed to prevent relapse have reported a reduction of the craving sensation and a decrease in the amount of cannabis consumed (101). Furthermore, several cannabis-related psychoeducation online platforms were launched recently, but the evidence on their effectiveness is limited, as they have rarely been properly evaluated with regard to the content quality (102). These results are particularly encouraging for some countries such as Canada (the first G-7 country to legalize cannabis), knowing that the profiles of cannabis users in this country are quite different from those of users of other substances, with most cannabis users being under the age of 20 and still in high school. This profile should influence the content of the treatment and the manner in which the treatment is offered (103). Many youths will wait years before deciding to seek professional help for their cannabis use. This is partly due to the stigma associated with seeking professional help, as well as the fact that it can take that long for youths to realize that their cannabis use might be a problem.

In alcohol-related problems, researchers using an application designed to provide support to people with alcoholism (Addiction—Comprehensive Health Enhancement Support System or A-CHESS) were able to partially predict the number of days of consumption based on the level of use of the application by the user (i.e., number of times spent on the application, number of pages viewed) (104). As in the case of the Stop-cannabis application presented above, the design of the A-CHESS application was based on the model of the self-determination theory. In trauma-related conditions, Possemato et al. (105) evaluated the effectiveness of an application (PTSD Coach) in reducing the symptoms associated with trauma in groups of veterans diagnosed with PTSD separated into two conditions (application with or without clinician assistance). The application has been associated with a decrease in symptoms under both conditions, with a significant benefit for the clinician-assisted condition. Other studies also report positive outcomes with the use of mobile applications for the management of different mood disorders (106–108).

Some recent research in mental health apps has evaluated the effectiveness of interventions that promote emotion regulation. To date, two applications were developed for a population with borderline personality disorder. EMOTEO, an application developed by a research team at the University of Geneva, was developed with the aim of providing emotional support during crises occurring between consultation sessions with the health care team (109). It allows the user to use mindfulness or distraction exercises according to the level of distress that has been reported initially using a distress scale ranging from 0 to 10 (i.e., mindfulness exercise when the tension is between 1 and 6 and distraction exercises when it is between 7 and 10—following the idea that users in intense emotional distress don’t have a lot of resources to focus on a more complex exercise). The user is invited to report his/her level of distress once again after completing the exercise. The levels of distress that have been reported over time are reported on a graphic accessible from the main menu. The application also allows a direct call to emergency services if the app user feels the need to make one (109). DBT Coach is an application designed to promote the implementation of new skills acquired in therapy in the natural environment of the individual. More specifically, it aims to help the user develop the competency of the opposite action, a technique taught in DBT that consists of acting in a way that neutralizes a negative overwhelming emotion (110). In order to suggest an opposite action adequate for the state of the individual, the application first asks the user to evaluate the intensity (on a scale of 0 to 10) and the quality of the emotion felt. After this information is entered, the program asks the individual whether or not he/she is ready to change the emotion. In the case of a positive response, the application will ask the user to indicate a specific action urge that he/she feels in connection with the reported emotion and will then provide a list of opposing actions that can be performed in response to this emotion. A negative answer will show windows where the user can indicate the “pros” and the “cons” to changing an emotion. Both applications have shown promising results in reducing the distress associated with a dysphoric mood, in reducing the “urgency to consume” (“craving”), and in the improvement of the depressive state (109, 110).

Mobile apps are increasingly recognized as effective tools for facilitating behavioral change (111). Their practical format facilitates the implementation of new strategies directly in the environment where their difficulties have emerged (5). Emotion regulation applications help individuals manage the different features of an emotional reaction when they occur in their environment. Following this principle, several mood-enhancing applications have been shown to be effective in reducing anxiety levels (e.g., 88, 112, 113). and improving depressive symptoms (e.g., 107, 108, 112, 114), two emotions known to increase the risk of relapse in substance use (115, 116). Applications could then potentially reduce the risk of relapse by aiming to stabilize the emotions associated with it.

### Challenges and Limitations

Issues of security and privacy of applications are a major barrier to the acceptability of mental health applications, particularly when it comes to the transmission of non-Internet information (117). Not only does the information collected have the potential to be detrimental to the individual; applications may record a substantial amount of data on the individual [e.g., visited environments that have been recorded through geo-location system (118)]. Studies report very few mental health applications with an adequate privacy policy or secure data transmission methods (119–121). Huckvale et al. (122) conducted a systematic assessment of the applications presented on the English National Health Service (NHS) website, a public health organization with a mandate to ensure compliance with data protection regulations in England (122). Applications were evaluated based on their compliance with data collection/transmission/storage guidelines, confidentiality, privacy policy content, and the adequacy of these policies for the application’s actual behavior. One of the main findings reported by the research team concerns the high variability of the security level of the applications. Only one-third of the apps available had a confidentiality policy, which, in most cases, was incomplete (e.g., no specification on the use of data by a third party or incomplete explanations of the process for requesting changes to registered data as allowed by law). The study also reports that a large number of applications stored and transmitted unencrypted personal information. In light of these findings, the researchers point out that enforcement accreditation by a government agency does not guarantee the security of the data that will be recorded and invite users to exercise caution. Some authors also link the lack of consistency in the security of applications to the fact that government regulations in this field are sometimes ambiguous—e.g., an application pretending to cure a condition will not be subject to the same level of evaluation as an application designed to “improve mood” (89). Unfortunately, policies protecting app users are not rigorously being applied today and are lacking information in some important areas such as the use of data by a third party (121). As a result, users are encouraged to exercise caution when choosing a mental health application (74).

The speed at which technology is evolving today makes it difficult to accommodate the empirical validation process, which always requires a certain amount of time (74). Moreover, several issues regarding the validation of EMA tools are still in need of attention. First, although the EMA is more suitable for capturing intra-individual differences over time, this is not always the case for recall-based questionnaires (75). One challenge in this situation would be to adapt and validate tools compatible with the characteristics of the EMA. Furthermore, very few studies have examined the psychometric properties of tools used to collect information on mobile phones. These properties could potentially differ from those used for more traditional questionnaires, because they are not always used in the same context—e.g., users could fill out the questionnaire on their phone while they perform other tasks at home (123). Finally, it is possible that the small number of items generally used in EMA tools may produce limited results where we only scratch the surface of a particular phenomenon.

It is important to mention that the use of mental health apps in a population presenting a substance use disorder carries a risk of cross-dependence (124). Several studies have found similarities between certain behaviors associated with the problematic use of the Internet (i.e., gambling, compulsive pornography viewing) and substance use problems, including 1) unsuccessful attempts to reduce or put an end to the problematic behavior, 2) concerns (feeling of craving) leading to problems of functioning, and 3) continuation of behavior despite deleterious effects (124–126). There is also evidence of an association between behavioral addiction (i.e., pathological gambling, compulsive buying, Internet addiction, work addiction, and exercise addiction) and problematic use of mobile phones in youth (127).

## Conclusion

The treatment of individuals with SMI is often long and complex, in part because of the important functioning impairments but also because of the clinical presentation. The clinical presentation is often complicated by the presence of many comorbidities, such as substance use disorders, which are more often the rule than the exception. As in other mental health conditions, individuals with SMI and comorbid substance misuse often face accessibility difficulties for treatments offered in mental health settings. The mobile phone could potentially be part of the solution to this issue by providing immediate access to strategies, when needed. Studies on this new form of intervention method report encouraging results for several psychiatric conditions. However, more research with comparison groups is needed to determine their clinical efficacy with greater certainty. In terms of intervention techniques, treatments aimed at improving emotion regulation have been shown to be effective for people with different diagnoses, including SMI and substance misuse.

In this perspective, an intervention promoting the development of emotional regulation skills for a population with comorbid SMI and substance misuse could potentially facilitate access to care and promote better management for those individuals. Our research team is now working on the development and evaluation of such an application, focusing on emotional regulation, that takes into account the specific characteristics of this population by applying the rules for designing applications for persons with cognitive deficits (95). The application for dual disorders, called Chill Time, includes a module dedicated to assessing distress and a module offering different intervention strategies primarily based on cognitive behavioral and third-wave strategies for emotion regulation. The application consists of 20 exercises divided into four categories: 1) cognitive, 2) emotional, 3) behavioral, and 4) spiritual strategies. Each of the strategies employed by the user is evaluated on its perceived usefulness once the exercise is completed. The application is currently under final development and validation. Should the results be positive, such an application may increase access to services and help link actual *in vivo* strategies with therapeutic outcomes, as assessed by clinicians. The assessment module includes a notification system that is similar to what can be observed with other social network apps; two alerts per day in total will be sent to the user. Once the notification is opened, the user will be redirected to the application, where he/she will be asked to rate his/her distress on a scale of three anchor points (i.e., good, neutral, not good). Should the response be neutral or negative, the user is redirected to a list of two exercises he/she could perform to emotionally self-regulate (stemming from two different categories). The two suggested exercises are chosen on the basis of the user’s past evaluations of the strategies’ usefulness (the exercises offered are initially randomly offered until the application has collected enough information on the preferences of the users).

Mobile applications have the potential to assist people with dual disorders in using strategies between treatment sessions without having to rely on their memory. Future studies are needed in order to demonstrate their efficacy and to offer profiles of emotional strategies used and their impact on substance misuse. Although multiple applications are being developed, only those with strong empirical support should be recommended and used.

## Author Contributions

AP did most of the writing and research. The other three authors (TL, SP, YK) contributed to the article by writing and editing the sections corresponding to their area of expertise in collaboration with the first author.

## Conflict of Interest Statement

One author (SP) is the holder of a grant from Otuska Pharmaceuticals unrelated to the topic of the current paper. The remaining authors declare that the research was conducted in the absence of any commercial or financial relationships that could be construed as a potential conflict of interest.
